# Synovial joints were present in the common ancestor of jawed fish but lacking in jawless fish

**DOI:** 10.1371/journal.pbio.3002990

**Published:** 2025-02-25

**Authors:** Neelima Sharma, Yara Haridy, Neil Shubin

**Affiliations:** Department of Organismal Biology and Anatomy, The University of Chicago, Chicago, Illinois, United States of America; University of California San Diego, UNITED STATES OF AMERICA

## Abstract

Synovial joints, characterized by reciprocally congruent and lubricated articular surfaces separated by a cavity, can simultaneously provide mobility and load bearing. Here, we study the early evolution of synovial joints by examining the morphological, genetic, and molecular features required for the development and function of the joints in elasmobranchs and cyclostomes. We show the presence of cavitated and articulated joints in the skeleton of elasmobranchs, such as the little skate (*Leucoraja erinacea*) and bamboo shark (*Chiloscyllium plagiosum*). However, our results do not support the presence of articular cavities between cartilaginous elements in cyclostomes such as sea lampreys (*Petromyozon marinus*) and hagfish (*Myxine glutinosa*). Immunostaining reveals the expression of lubrication-related proteoglycans like aggrecan and glycoproteins such as hyaluronic acid receptor (CD44) at the articular surfaces in little skates. Analysis of joint development in little skate embryos shows the expression of growth differentiation factor-5 (Gdf5) and *β*-catenin at the joint interzones like tetrapods. Muscle paralysis in little skate embryos leads to joint fusion, suggesting that muscle activity is necessary for the formation of synovial cavity and development of normal articular surfaces, in a manner similar to zebrafish and tetrapods. Together, these data suggest that synovial joints originated in the common ancestor of extant gnathostomes. A review of fossils from the extinct clades along the gnathostome stem suggests that joints with reciprocally articulating surfaces arose in the dermal skeleton of the common ancestor of all jawed vertebrates. Synovial joints in cartilaginous tissue were a subsequent gnathostome innovation.

## Abstract 2

Synovial joints are characterized by lubricated articular surfaces separated by a cavity, providing mobility and load-bearing, but when did they evolve? This comparative and developmental study reveals that stable, mobile and lubricated joints were present in the common ancestor of jawed fishes but lacking in jawless ones.

## Author summary

We owe much of our mobility and agility to synovial joints. Characterized by a lubricated joint cavity between bony elements, synovial joints function by the relative sliding of bones against each other. They allow an extensive range of motion and heightened stability compared to fused or cartilaginous joints that function by bending. Using histological and protein expression analysis, we show that reciprocally articulated, cavitated, and lubricated joints are present in chondrichthyans such as skates but not in cyclostomes such as lamprey and hagfish. Moreover, the little skate joints express proteins from the TGF-*β* and Wnt genetic regulatory pathways, which are known to assist joint development in zebrafish and tetrapods. Little skate joints rely on muscle contraction to form the joint cavity like tetrapods. Thus, our results show that synovial joints are present in chondrichthyans but likely not in cyclostomes, and we conclude that synovial joints originated in the common ancestor of jawed vertebrates. Furthermore, we review the fossil taxa along the gnathostome stem to show that cavitated joints that function by relative sliding of articulating surfaces originated at the common ancestor of all gnathostomes. Our study has implications for understanding the early evolution and diversification of synovial joints in vertebrates.

## Introduction

Synovial joints provide stability, load-bearing capacity, an extensive range of motion, and better control of rotational degrees of freedom than other kinds of joints in the vertebrate skeleton [1]. Comparative and molecular analyses show that zebrafish, gar, lungfish, and tetrapods exhibit lubricated and cavitated joints, suggesting an origin of synovial joints at the common ancestor of the osteichthyan clade [2]. However, Davies [3] described synovial-like morphology in skates (multiple species of *Raia*), and Haines [4] noted their presence in the jaws of *Chimaera monstrosa* using histological analyses. If true, these observations suggest that the origin of synovial joints predated the common ancestor of osteichthyan fish. Here, we aim to analyze the joints of cyclostomes and elasmobranchs using developmental and molecular analyses to confirm earlier reports of potential synovial morphology in elasmobranchs and to understand the morphology of joints in cyclostomes. Moreover, we review and analyze the joint morphology in the fossil record of early vertebrates to assess the diversity and scenarios for the origins of these joints.

Synovial joints are characterized by a lubricated joint cavity between the articulating bones [5]. A thin layer of hyaline articular cartilage covers the articular surfaces in tetrapods [6] and finned bony fishes [2] and is separated into superficial, transitional, middle, and calcified zones. Shared expression of chondrocytes and extracellular matrix (ECM) proteins such as collagen-I and collagen-II in the developing joints [7] and the expression of proteoglycans such as lubricin (Prg4) and aggrecan (Acan), glycosaminoglycans (GAGs) such as hyaluronic acid, and glycoproteins such as hyaluronic acid receptor (CD44) at the articular regions suggest synovial joints [6,8]. Lubricating proteins such as lubricin and aggrecan in the articular cartilage or synovial cavity provide a near frictionless joint motion by providing articular boundary lubrication under loads and prevent the chondrocytes in the articular region from damage during compression [9,10].

The morphogenetic and molecular mechanisms for the development of synovial joints typically differ based on whether they occur in the endochondral skeleton, most notably the appendicular skeleton, or in the dermal skeleton, such as the bony cranium and mammalian jaw [11,12]. In the appendicular skeleton, the development of synovial joints begins with the condensation of an uninterrupted mesenchyme region and the presumptive site of joint cavitation called the interzone. The joint interzone undergoes cavitation, and the segmenting mesenchyme differentiates into chondrocytes of the primary cartilage [11,13] to form two independent articulating elements. The joint interzone cells express growth and differentiation factor 5 (*Gdf5*) from the transforming growth factor-*β* (TGF-*β*) superfamily and *β*-catenin from the canonical Wnt pathway and differentiate into tissues forming articular cartilage, ligaments, and the joint capsule [14–17]. Cyclic muscle contraction is necessary for joint cavitation [14,15,18,19], such as shown in chicken and mice limbs [19–24]. In mice, the extension and flexion of the appendicular skeleton due to muscle contraction forms and expands the microcavities in the interzone to give rise to the synovial cavity [21]. Without muscle activity, the joint cavity does not form and the joints remain fused in mice [20], leads to a weaker Wnt-signaling in the joint cells and abnormal articular surface morphology in zebrafish [22].

The formation of synovial joints in the dermal bones differs from endochondral bones in both the morphogenetic processes and developmental genetic mechanisms [12,25]. The process does not involve contiguous cartilaginous elements or joint interzones. Instead, separate mesenchymal condensations give rise to individual bony segments through intramembranous or perichondral ossification [12]. The articular cartilage and other joint connective tissue such as the ligaments and joint capsule appear from the cells between the articulating bones. The joint cells do not express *Gdf5* but express molecules related to *Gli2* and *Hedgehog* signaling, as shown in mouse temporomandibular joint [25].

Here, we study the joints in the cartilaginous skeleton of cyclostomes and elasmobranchs to extend the phylogenetic range of our understanding of synovial joints and to infer the origin of synovial joints in the phylogenetic tree of extant vertebrates [3,4]. Using histological and molecular techniques, we analyze the morphology and development of the joints of elasmobranchs such as the little skate (*Leucoraja erinacea*) and bamboo shark (*Chiloscyllium plagiosum*), and cyclostomes such as lampreys (*Petromyozon marinus* and *Ichthyomyzon bdellium*) and hagfish (*Myxine glutinosa*). Furthermore, early vertebrate evolution is composed of multiple extinct lineages along the gnathostome stem [26]. We use μCT and histological techniques to assess the earliest joints in this part of the vertebrate phylogenetic tree.

## Results and discussion

### Cavitated joint articulations are present in elasmobranchs but not in cyclostomes

**Fig 1:**
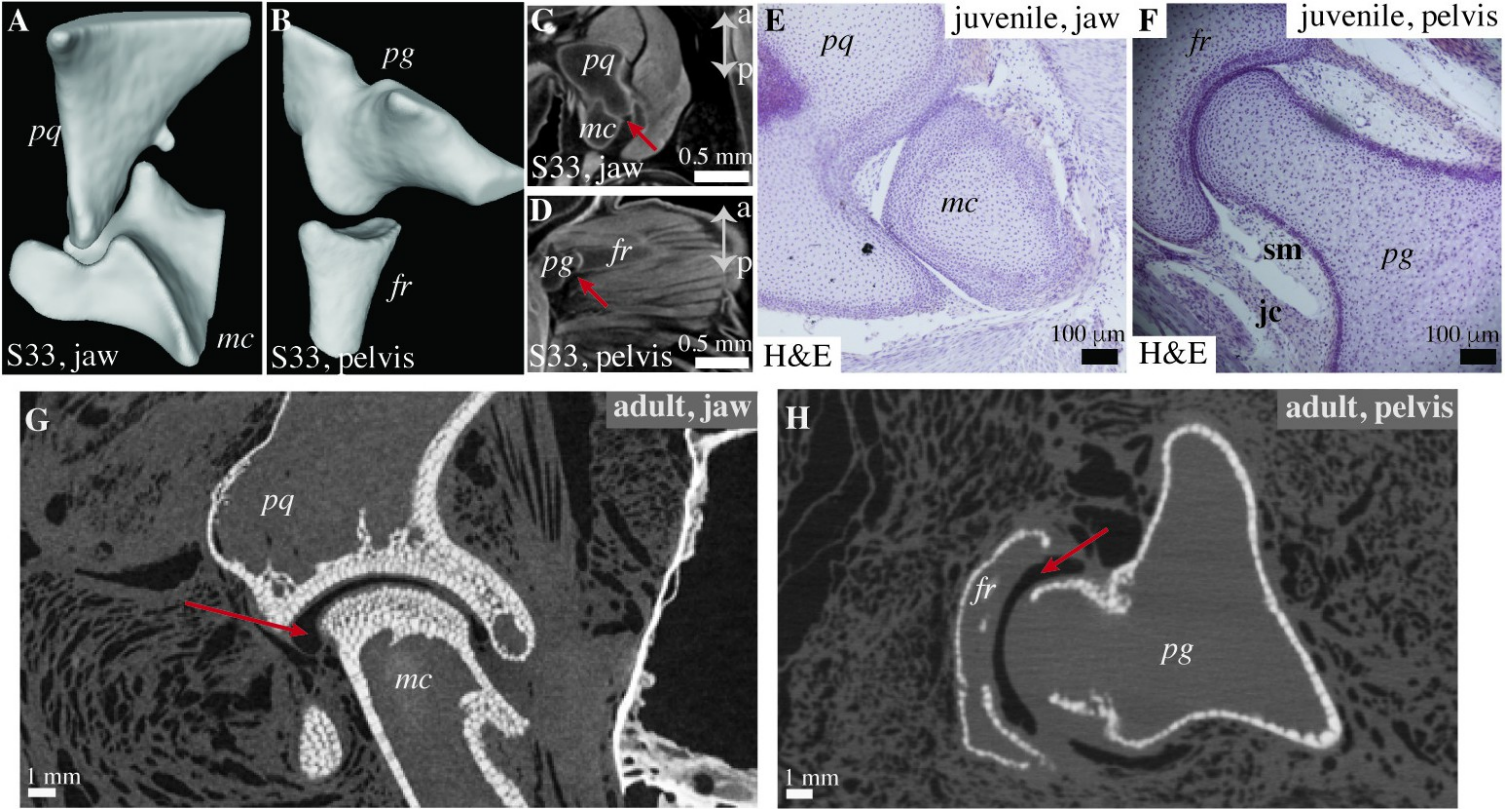
μCT scans and histology show cavitated and reciprocal articulations in embryonic and juvenile little skates (*Leucoraja erinacea*). (A, B) 3D reconstruction of the cartilaginous skeleton from the μCT scan of the little skate embryo (stage 33) shows articulated joints with reciprocal surface geometries in the jaw (A) and the pelvis (B). (C, D) Virtual sections from the μCT scans of the little skate embryo (stage 33) display the articulations (red arrow) in the jaw (C) between the palatoquadrate (*pq*) and the meckel’s cartilage (*mc*), and the pelvic fin (D) between the pelvic girdle (*pg*) and the first ray (*fr*). (E, F) Histochemical (HE) staining shows the presence of cavitated joints in the jaw (E) and the pelvis (F) of the juvenile skate. (G, H) Virtual sections from the μCT scan of the adult little skate display reciprocal articular surfaces and joint cavities (red arrows) in the jaw (G) and the pelvis (H). The underlying data can be found at Morphosource ID: 000623009 for A and C, at Morphosource ID: 000623081 for B and D. a, anterior; p, posterior; pq, palatoquadrate; mc, meckel’s cartilage; pg, pelvic girdle; fr, fin ray; sm, synovial membrane; jc, joint capsule.

We analyze the joint morphology of little skates (*Leucoraja erinacea*), sea lamprey (*Petromyozon marinus*), and hagfish (*Myxine glutinosa*) using a variety of techniques. Contrast-enhanced μCT scanning reveals the presence of reciprocal articulating elements separated by a joint cavity in the skeleton of the late-stage (stage 33) embryo of the little skate [27] (Fig 1A–1D). Histological analyses using hematoxylin and eosin (HE) reveal joint cavities lined by synovial membrane and joint capsule in the jaw and pelvis of juvenile skates (Fig 1E and 1F). Analysis of the virtual sections from the μCT scan of an adult little skate shows that the reciprocal articulating segments in both the pelvis and the jaw are separated by a joint cavity, indicated by the darker grayscale value representing empty space (Fig 1G and 1H).

**Fig 2:**
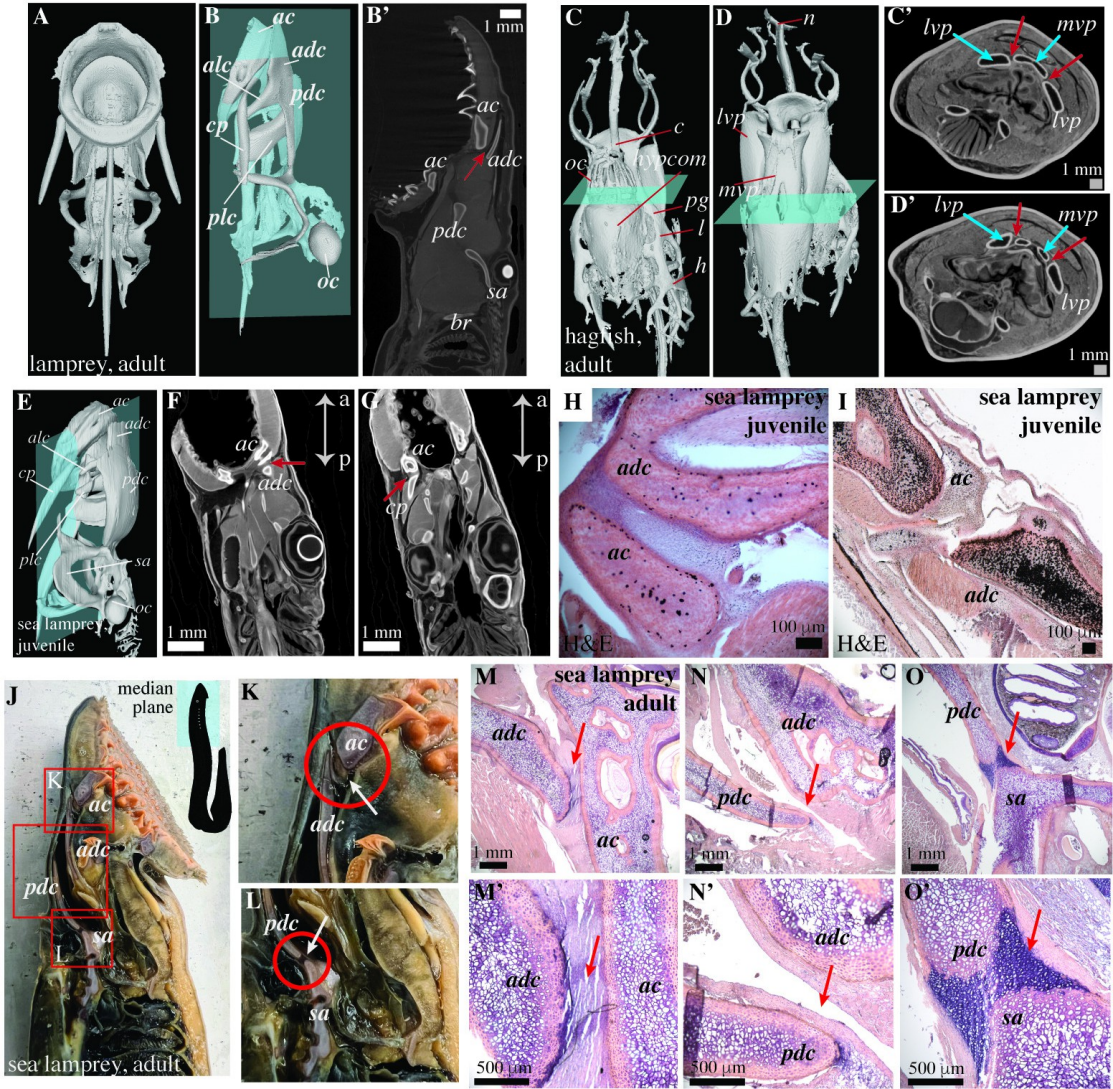
μCT scans and histology show non-cavitated and non-reciprocal joints in lampreys (*Ichthyomyzon bdellium* and *Petromyozon marinus*) and hagfish (*Myxine glutinosa*). (A, B) 3D reconstruction of the cartilaginous skeleton from the contrast-enhanced μCT scan of an adult lamprey (*Ichthyomyzon bdellium*) in the ventral (A) and lateral (B) view. The blue plane in B highlights the section shown in B’. (B’) Virtual section obtained from the μCT scan of the adult lamprey (*Ichthyomyzon bdellium*) displays non-reciprocal cartilaginous elements in the chondrocranium and the absence of any joint cavities, as evidenced by the presence of grayscale values between *ac* and *adc* that depict soft tissue (red arrow). (C, D) 3D reconstruction of the cartilaginous skeleton from the contrast-enhanced μCT scan of an adult hagfish (*Myxine glutinosa*) in the dorsal (C) and ventral (D) views. The blue planes highlight the sections shown in C’ and D’. (C’, D’) Virtual sections from the μCT scan of the adult hagfish display non-reciprocal cartilage elements *mvp* and *lvp* in the chondrocranium and the absence of joint cavities (red arrows). (E–G) 3D reconstruction of the cartilaginous skeleton from the μCT scan of a juvenile sea lamprey (*Petromyozon marinus*) in a lateral view. The blue plane shows the orientation of virtual sections in F, G that display non-reciprocal cartilaginous elements and an absence of joint cavities. (H, I) Histochemical (HE) staining shows the presence of uncavitated joints between the cartilaginous elements of the chondrocranium of the juvenile sea lamprey. (J–L) Dissection of an adult sea lamprey (*Petromyozon marinus*) reveals the joints in the chondrocranium in the median plane. Red boxes represent regions where the cartilaginous elements form neighboring pairs. White arrows in the zoomed-in photographs K and L indicate the cartilaginous joints between *ac* and *adc* (K), and *pdc* and *sa* (L). (M–O’) H&E staining demonstrates that the joints between the neighboring cartilaginous joints in sea lamprey do not exhibit a synovial morphology. The data underlying panels C–D’ can be found at Morphosource ID: 000661650 and for E–G at Morphosource ID: 000623131. Abbreviations for lamprey are based on Ayers [28]: *ac*, annular cartilage; *adc*, anterior dorsal cartilage; *pdc*, posterior dorsal cartilage; *sa*, subocular arch; *oc*, otic capsule; *alc*, anterior lateral cartilage; *cp*, copula; *plc*, posterior lateral cartilage; *br*, brachial basket. Abbreviations for hagfish: *c*, cornual cartilage; *oc*, olfactory capsule; *d*, dental plate; *l*, lateral labial cartilage; *pq*, ptergo-quadrate bar, *lvp*, latero-rostral part of basal plate; *mvp*, medio-rostral part of basal plate, *h*, hyoid; *hypcom*, hypophyseal commissure.

To understand if the absence of cavitated joints is a general trait in vertebrates, we sampled cyclostomes, including their adult stages. Adult lamprey (*Ichthyomyzon bdellium*) and adult hagfish (*Myxine glutinosa*) (Fig 2A–2D’) have neighboring cartilaginous segments separated by region with grayscale values that do not suggest the presence of a cavity (Figs 2B’, 2C’ and 2D’). Similarly, no joint cavity is observed between the neighboring cartilaginous segments in the chondrocranium in the juvenile sea lamprey (*Petromyozon marinus*), as evidenced by the presence of grayscale values in the virtual sagittal sections (Fig 2E–2G) and histological analyses (Fig 2H and 2I). Dissection of an adult sea lamprey (*Petromyozon marinus*) to reveal its chondrocranium in the median plane (Fig 2J) shows that the cartilaginous elements *ac* and *adc*, and *pdc* and *sa* are continuous and do not exhibit a joint cavity (Fig 2J–2L). Although a cavity exists between *adc* and *pdc*, the anterior and posterior edges of these segments are embedded in surrounding tissue and unable to engage in relative sliding (Fig 2J), suggesting that they do not articulate with each other. Finally, histological analyses of an adult sea lamprey also support an absence of reciprocal articular surfaces and joint cavities (Fig 2M–2O’). The segments are either connected by muscles (Fig 2M and 2M’), connective tissue (Fig 2N and 2N’), or regions that resemble qualitatively different cartilage compared to the flanking elements (Fig 2O and 2O’). Furthermore, reciprocal articular shapes, joint cavities, and synovial membranes are not observed.

**Fig 3:**
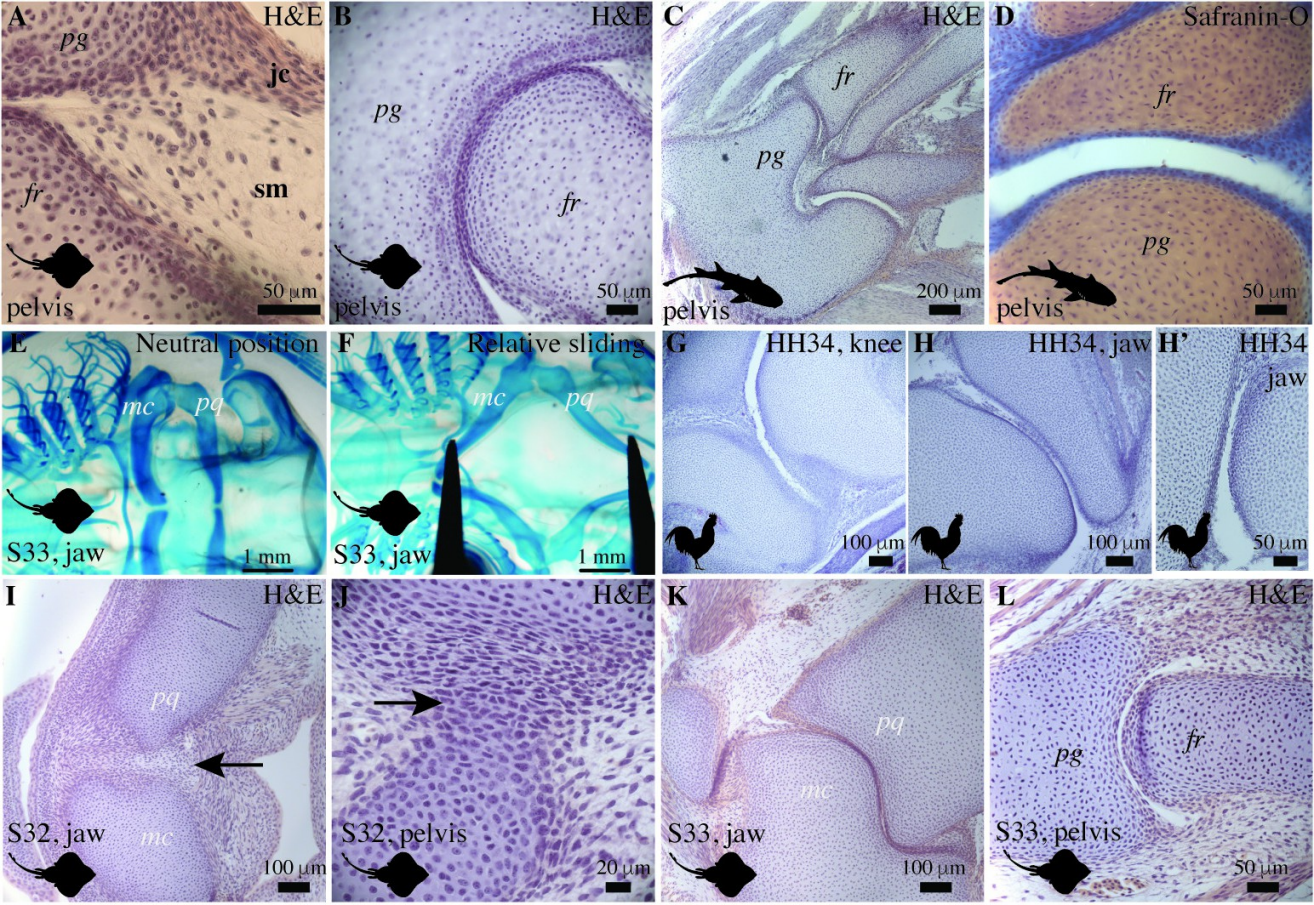
Comparison of the little skate, bamboo shark, and chicken joints exhibits similarities between elasmobranch and tetrapod joints. (A, B) HE staining of a little skate pelvis (juvenile) shows that individual cartilage elements in the pelvic fin are separated by a cavity. A distinct synovial membrane and joint capsule are present. The joint capsule is in continuity with the perichondrium (A), and the articular surfaces have flattened chondrocytes (B). (C, D) HE and safranin-O staining of the sections of a bamboo shark (belonging to the group selachians) demonstrates the presence of cavitated joints and articulations lined by flattened chondrocytes in the pelvis. (E, F) Manual movement of the jaw in a whole mount alcian and alizarin red stained specimen of the little skate (stage 33) demonstrates relative sliding of the articular surfaces (F) compared to the neutral position (E). (G, H, H’) HE staining shows that the cavitated joints in the knee and the jaw of an embryonic chicken (HH34) exhibits cavitated joints and flattened chondrocytes. (I, J) HE staining of the jaw (I) and the pelvic joint (J) of the little skate (stage 32) display uncavitated joints and the interzone (black arrows). (K, L) HE staining of the jaw (K) and the pelvic joint (L) of the little skate (stage 33) display cavitated joints. sm, synovial membrance; jc, joint capsule; pq, palatoquadrate; mc, meckel’s cartilage; pg, pelvic girdle; fr, fin ray.

The joint cavity in the pelvis of juvenile little skates is lined by a membrane characterized by a relatively acellular connective tissue containing folds and an outer layer characterized by fibrous tissue connecting the two cartilaginous elements (Fig 3A). The structure of the lining containing crimpled folds facing the joint cavity and fibrous capsule lining resembles the synovial membrane and joint capsule found in tetrapods [29]. In little skates, a high density of flattened articular chondrocytes line the articular cavity, and hypertrophic chondrocytes are present in the subarticular regions (Fig 3B). In a fixed whole-mount skeleton of the little skate (stage 33) stained with alcian blue and alizarin red, manual opening and closing of the jaw shows relative sliding of the articular surfaces, similar to how synovial joints operate (Fig 3E and 3F). The morphology of skate joints is similar to the cavitated joints of a chicken embryo (Fig 3G, 3H, and 3H’) that are known to exhibit relative sliding. Analysis of the developmental stages reveals that the embryonic skate at stage 32 has uncavitated joints (Fig 3I and 3J), whereas stage 33 reveals cavitated ones (Fig 3K and 3L) in the jaw and the pelvis. Thus, little skate jaw and pelvic joints form by cavitation in the uninterrupted cartilage, similar to endochondral bone.

To understand whether cavitated joints are a consistent feature in elasmobranchs, we analyzed the joints of bamboo sharks (*Chiloscyllium plagiosum*), a member of the selachian clade, a sister group of batoids. In the pelvic joints of bamboo sharks, we observed cavitated joints with flattened chondrocytes lining the articular surfaces, revealing a synovial-like morphology in the pelvic joints (Fig 3C and 3D). The presence of reciprocally cavitated articular surfaces containing flattened articular chondrocytes lined by synovium is evidence of synovial-like morphology in little skates and bamboo sharks. Morphological analyses suggest that cavitated synovial-like joints are a conserved feature in chondrichthyans, actinopterygians, and sarcopterygians (including tetrapods). This survey of extant taxa would imply that synovial-like joints are a feature of the last common ancestor of gnathostomes.

### Collagen-II forms the primary component of subarticular cartilage while Collagen-I is limited to the articular surface in developing little skate joints

**Fig 4:**
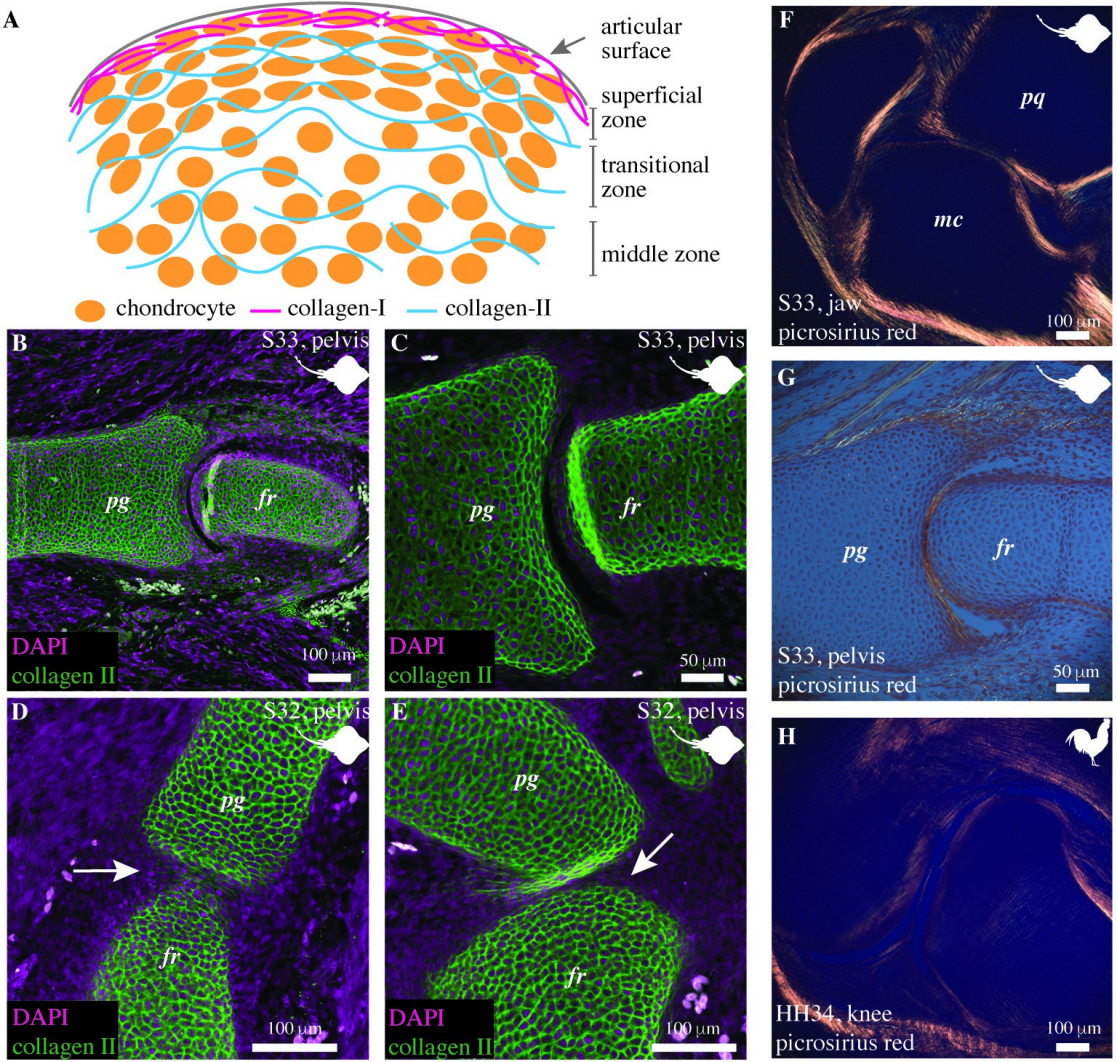
The articular region of embryonic little skates is composed of collagen-I and collagen-II. (A) A pictorial representation of the organization of chondrocytes and ECM at the articular and the subarticular surfaces of a developing synovial joint. The perichondrium near the superficial zone displays collagen-I, the transition zone displays collagen-II parallel to the articular surface, and the middle zone displays a nonspecific arrangement of collagen-II fibers. (B, C) The cavitated pelvic fin joints of the little skate embryos (stage 33) express collagen-II parallel to the articular surface and are randomly distributed in the subarticular region. (D, E) The uncavitated pelvic fin joints of the little skate embryos (stage 32) display an interzone (displayed by arrows) with an extracellular matrix containing collagen-II. The ECM shows signs of being mechanically strained. (F, G) Picrosirius red staining is used for visualizing collagen-I in orange/yellow and suggests that collagen-I is present at the articular surface perichondrium in the jaw (F) and the pelvic joint (G) of the little skate (stage 33). (H) Picrosirius staining helps visualize collagen-I in the perichondrium lining the articular surfaces of the knee joint of a chicken embryo (HH34). pq, palatoquadrate; mc, meckel’s cartilage; pg, pelvic girdle; fr, fin ray.

Developing tetrapod articular cartilage is composed of collagen-II and lined by perichondrium composed of collagen-I (Fig 4A) [7,30–32]. Previous studies show that collagen-II forms the bulk of the cartilaginous skeleton in little skates [33]. Here, we investigate the composition of little skate articular cartilage and compare it with tetrapod articular cartilage. We analyze the organization of the extracellular matrix in the articular and the subarticular cartilage in little skates by immunostaining for collagen-II and using picrosirius staining for collagen-I. Our data reveal that the collagen-II is present in the articular surfaces, as well as the subarticular cartilage in the cavitated pelvic joints of the skate embryo (stage 33, Figs 4B, 4C, and S1). In the uncavitated joints of embryonic stage 32 of the little skate, we observe that collagen-II is present in the interzone. However, the interzone collagen is less dense than the presumptive articular surfaces and also shows signatures of being mechanically strained (Figs 4D, 4E, and S1), likely from cyclic muscle contraction [21]. Picrosirius red staining is used as an indirect method to locate the distribution of collagen-I. Visualizing the staining sections with a polarizer suggests that collagen-I is present in the joint capsules of the jaw and pelvic joints of skate embryos (stage 33) and in the perichondrium lining the articular surfaces (Fig 4F and 4G). The arrangement of collagen-I is similar to the developing knee joint in tetrapods like embryonic chicks (Fig 4H).

### Sea lampreys and little skates display glycosaminoglycans and proteoglycans at the articular surfaces in a pattern similar to tetrapods

**Fig 5:**
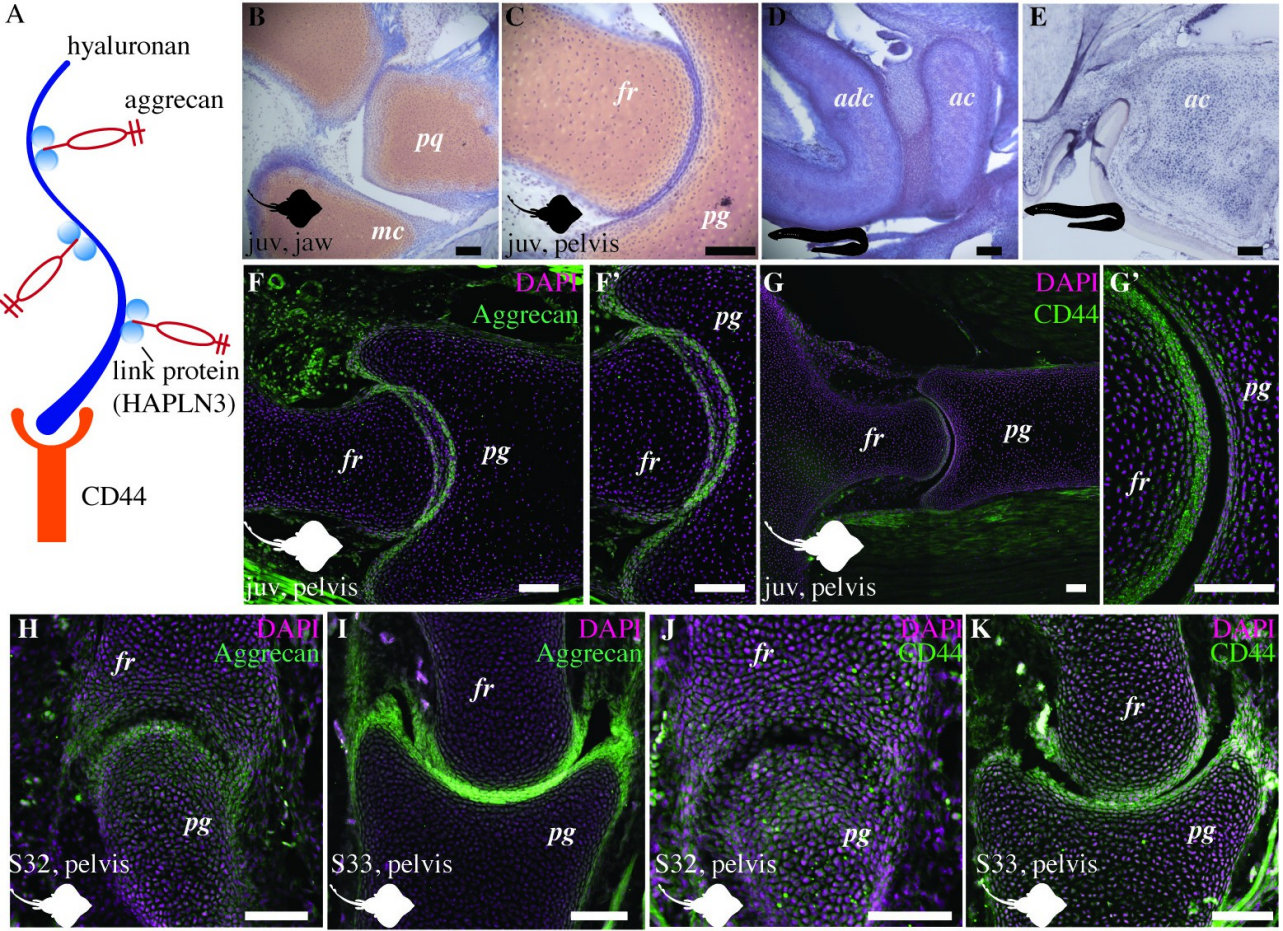
Little skate and the sea lamprey cartilage displays GAGs, proteoglycans, and glycoproteins. (A) A pictorial representation of the interaction between GAGs such as hyaluronan, link proteins such as HAPLN3, and proteoglycans such as aggrecan. (B–D) Safranin-O staining shows the distribution of glycosaminoglycans in the articular cartilage of the jaw (B) and pelvis (C) of the embryonic little skate (stage 33), and in the pharyngeal cartilage of the sea lamprey (D). (E) In situ hybridization reveals that the cartilaginous skeleton of the juvenile sea lamprey expresses the proteoglycan linking protein, HAPLN3. (F, F’) Aggrecan is expressed in the pelvic joint of juvenile skate in the articular cartilage and the synovial membrane. (G, G’) Hyaluronic acid receptor CD44 is expressed at the articular surfaces and the subarticular regions of the pelvic joints in the juvenile skate. (H, I) The little skate expresses aggrecan at the interzone as well as in the subarticular cartilage in the uncavitated stage 32 (H) and cavitated stage 33 (I) embryos. (J, K) The little skate expresses CD44 at the interzone and in the subarticular cartilage in the uncavitated stage 32 (J) and cavitated stage 33 embryos (K). juv, juvenile; pq, palatoquadrate; mc, meckel’s cartilage; pg, pelvic girdle; fr, fin ray; adc, anterior dorsal cartilage; ac, annular cartilage. Scale bars: 100 μm.

Glycosaminoglycans (GAGs) such as hyaluronan, chondroitin sulfate, and keratan sulfate are polyanionic extracellular matrix molecules found in the articular cartilage of tetrapods that function to keep the joint tissue hydrated by attracting water and aiding its shock absorption capacity [1]. GAGs like hyaluronan covalently attach to proteoglycans like aggrecan [8] and lubricin [10] using link proteins like hyaluronan and proteoglycan link protein HAPLN3 to form proteoglycan aggregates that are critical for lubrication [8,34,35] (Fig 5A). The aggregates organize and stabilize the interaction between hyaluronic acid and the extracellular matrix [36] and improve the shear and compressive load-bearing capacity of the cartilage [8,37,38]. Hyaluronan also plays a role in synovial cavity formation during development [39–42]. Previous studies have used biochemical assays to show the presence of GAGs in the skate cartilage [43] and have also demonstrated proteoglycan expression in the vertebral cartilage of chondrichthyans [44,45], notochord sheath of lampreys [46], and cartilage of lamprey embryos and larvae [47]. Here, we study the distribution of GAGs and proteoglycans in the joints of the cartilaginous skeleton of the sea lampreys and little skates.

Using safranin-O staining, we observe that glycosaminoglycans (GAGs) are present in the articular cartilage of juvenile little skates (Fig 5B and 5C). In the jaw and pelvic joints of the little skate, the amount of GAGs is lower at the articular surface compared to the deeper cartilage (Fig 5B and 5C), similar to tetrapods. Sea lamprey cartilage also has GAGs throughout the skeleton but primarily concentrated in the middle of the cartilage elements (Fig 5D). Using mRNA in situ hybridization, we observe that juvenile sea lamprey expresses hyaluronan and proteoglycan link protein (HAPLN3) throughout the cartilage skeleton (Fig 5E). Immunostaining reveals that the proteoglycan aggrecan is expressed in the articular cartilage in juvenile and embryonic skates (Figs 5F, 5F’, 5H, 5I, and S2). Immunostaining for CD44, the principal cell surface glycoprotein that acts as a receptor for hyaluronic acid [40], shows that it is expressed at the articular surfaces as well as in the subarticular regions, albeit at a lower amount, in the juvenile skate (Figs 5G, 5G’, and S3). Furthermore, both aggrecan and CD44 are also expressed in the synovial membrane, consistent with their expression in tetrapods [29]. The expression of proteoglycans and glycoproteins in juvenile chondrichthyans is consistent with their role in articular surface lubrication.

The little skates from embryonic stage 32 with uncavitated joints express aggrecan throughout the cartilaginous skeleton (Fig 5H), as shown previously [33], whereas stage 33 shows a more constrained expression at the articular surfaces compared to the bulk of the cartilage (Fig 5I). Immunostaining for CD44 reveals its expression in the interzone as well as the bulk cartilage of the joints of stage 32 little skate (Fig 5J), and a more constrained expression at the articular surfaces in the cavitated joints of stage 33 little skate (Fig 5K). Although aggrecan seems more localized in the articular cartilage in juvenile skates (Fig 5F), CD44 remains expressed in the bulk of the cartilage (Fig 5G). Whether adult little skates exhibit a more localized expression of CD44 at the articular surfaces like tetrapods [39] remains unknown. The expression of proteoglycans and glycoproteins in the joints of embryonic skates is consistent with their role in articular surface development.

### Key proteins from TGF-*β* and Wnt-signaling pathway are expressed during joint development in little skates

TGF-*β* and Wnt-signaling are required for joint morphogenesis in the endochondral bones of tetrapods. We examine whether joint morphogenesis in the little skate embryos is associated with the expression of proteins from these pathways. Studies show that little skates express *Gdf5* from the TGF-*β* superfamily in the intermediate region of the pharyngeal arches [48]. However, whether *Gdf5* is expressed at the joint interzones in little skates and if other developmental similarities exist between chondrichthyan and osteichthyan joints remains unclear.

**Fig 6:**
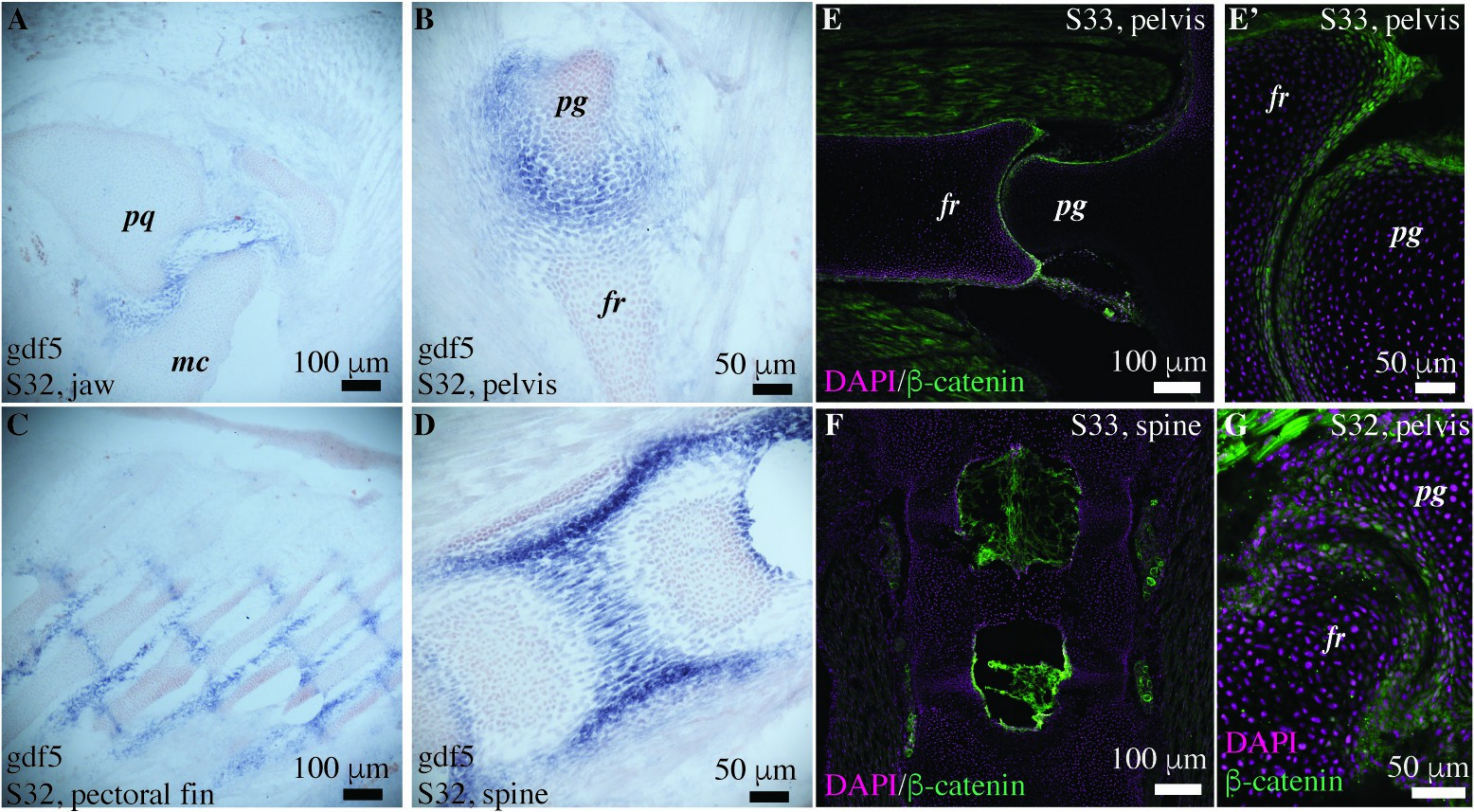
TGF-*β* and Wnt signaling are present during joint morphogenesis in the embryonic little skates. (A–D) In situ hybridization shows that growth differentiation factor 5 (*Gdf5*) is expressed at the joint interzones in the embryonic little skate at stage 32. The expression is seen at the interzones of the jaw (A), pelvis (B), and pectoral fin (C) that develop to form a joint cavity, and also in the intervertebral discs (D) that do not cavitate. (E, F) Immunostaining shows the expression of *β*-catenin in stage 33 embryos of the little skate at the articular surfaces of the pelvic joint (E, E’), but not in the intervertebral joints (F). (G) The uncavitated pelvic joint of the little skate (stage 32) expresses *β*-catenin at the articular surfaces. pq, palatoquadrate; mc, meckel’s cartilage; pg, pelvic girdle; fr, fin ray.

Using in situ hybridization, we observe that the embryonic little skate (stage 32) expresses *Gdf5* at joint interzones in the jaw, pelvis, and pectoral fin (Fig 6A–6C). *Gdf5* is also expressed in the non-cavitating intervertebral joints (Fig 6D). Immunostaining reveals that the pelvic joint of the little skate belonging to stage 32 and stage 33 express *β*-catenin at the articular surfaces (Figs 6E, 6E’, 6G, and S4) but the expression is not noticeable in the intervertebral discs (Fig 6F). The expression of *Gdf5* and *β*-catenin in the interzone cells is consistent with the later differentiation of the interzone into joint tissues such as articular cartilage, joint capsule, and ligaments [49]. In the cavitated joints of stage 33, the expression of *β*-catenin is also consistent with its role in specifying the superficial zone of the articular cartilage and the lubricating properties of the articular cartilage [50].

### Muscle contraction is essential for joint cavitation in the embryos of little skates

Muscle activity is necessary to develop a cavitated synovial morphology in the appendicular skeleton of tetrapods such as chick and mouse [19,20,51]. In zebrafish, the role of muscle activity on skeletal morphogenesis has predominantly been studied in the jaw, where the absence of muscle activity leads to stunted skeletal growth and abnormal chondrocyte morphology [22,23]. Wnt14-signaling is mechanosensitive in zebrafish [22] and plays a role in the downstream regulation of hyaluronic acid receptor (CD44) expression at the articular surfaces in chick embryos [14]. Furthermore, the mechanosensitive secretion of hyaluronic acid [52] plays a role in the process of joint cavitation in chick embryos [41,42]. Here, we test the effect of muscle contraction on joint morphology and developmental signaling in the joints of the little skates by inducing paralysis.

**Fig 7:**
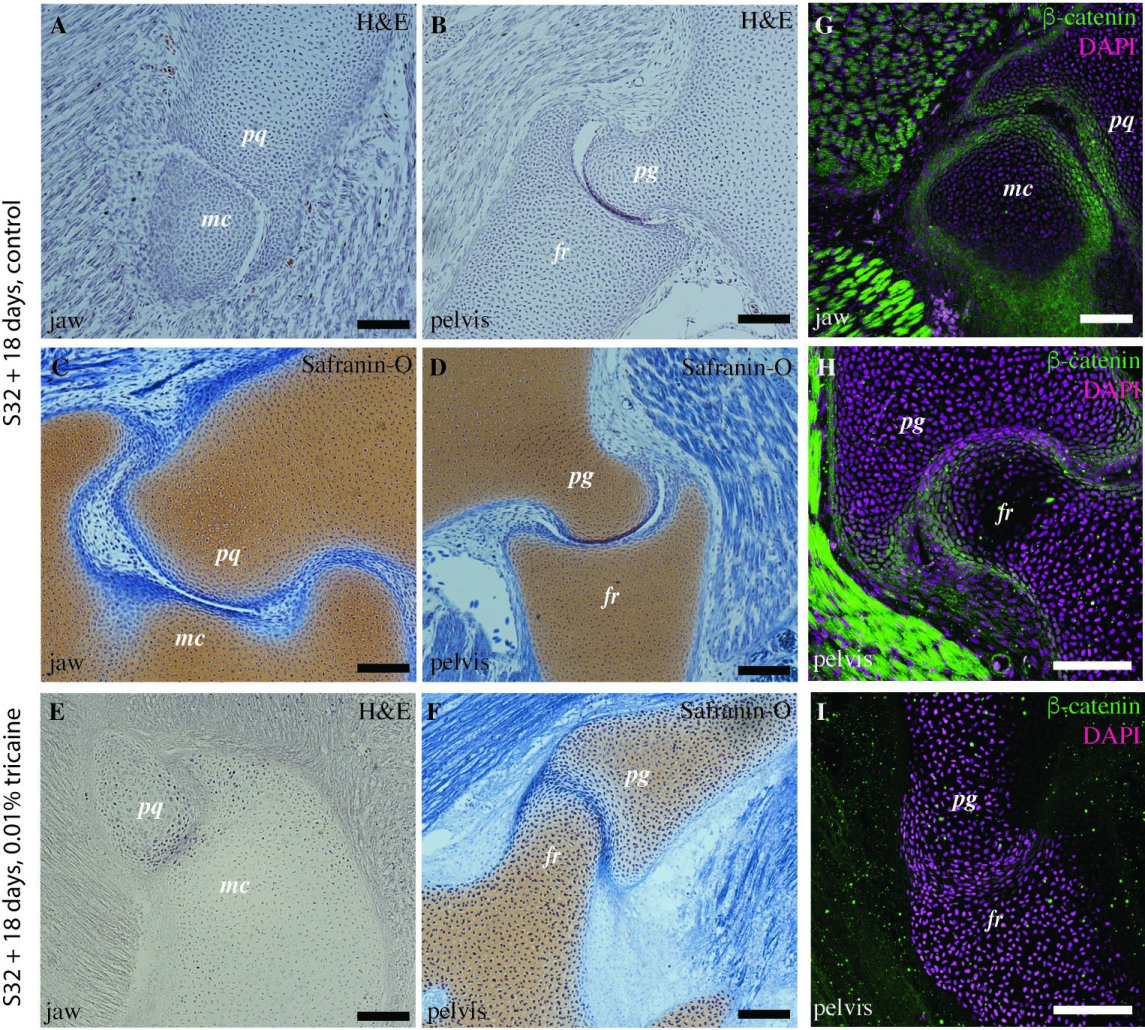
Muscle paralysis leads to joint fusion and impairs *β*-catenin signaling in the embryonic little skates. (A–D) HE and Safranin-O staining of the little skate (stage 32) control embryos raised for 18 days in seawater show that the joints are fully cavitated in the jaw (A, C) and the pelvis (B, D) with distinct joint cavities and synovial membrane. (E, F) HE and safranin-O staining of the little skate (stage 32) embryos raised in seawater infused with tricaine mesylate to induce paralysis shows fused and uncavitated joints with an interzone filled with cells and undefined articular regions compared to the control embryos. (G–I) Immunostaining for *β*-catenin in the jaw (G) and pelvis (H) of stage 32 little skate embryos shows expression in the articular cartilage of both the control and the paralyzed embryos, but a comparatively uncharacteristic and lower expression in the pelvis of the paralyzed embryos (I). pq, palatoquadrate; mc, meckel’s cartilage; pg, pelvic girdle; fr, fin ray. Scale bars: 100 μm.

We paralyzed stage 32 embryonic little skates for 18 days using low dosages of anesthetic tricaine mesylate (MS-222) and compared their joint morphogenesis with identically aged non-paralyzed control embryos. Histological and immunostaining comparison of paralyzed with the control stage 32 embryos reveal that control animals exhibit cavitated joints (Fig 7A–7D) and the identically aged but paralyzed embryos lack a clear joint cavity (Fig 7E and 7F). Flattened chondrocytes with a distinct orientation line the articular surfaces of control embryos, but the presumptive articular surfaces of paralyzed embryos lack any regular cell orientation (Fig 7A–7F). We observe that the control embryos express higher amounts of *β*-catenin in the chondrocytes at the articular surface than the paralyzed counterparts (Fig 7G–7I). Our investigations on the development of joints in little skates suggest that muscle contraction is necessary for cavitating the interzones of the jaw and pelvis to form a joint cavity. This mechanism is similar to that observed during joint morphogenesis in the endochondral skeleton of tetrapods such as chicks and mice [19,20,51].

### Analysis of fossils suggests that antiarchs had cavitated joints

**Fig 8:**
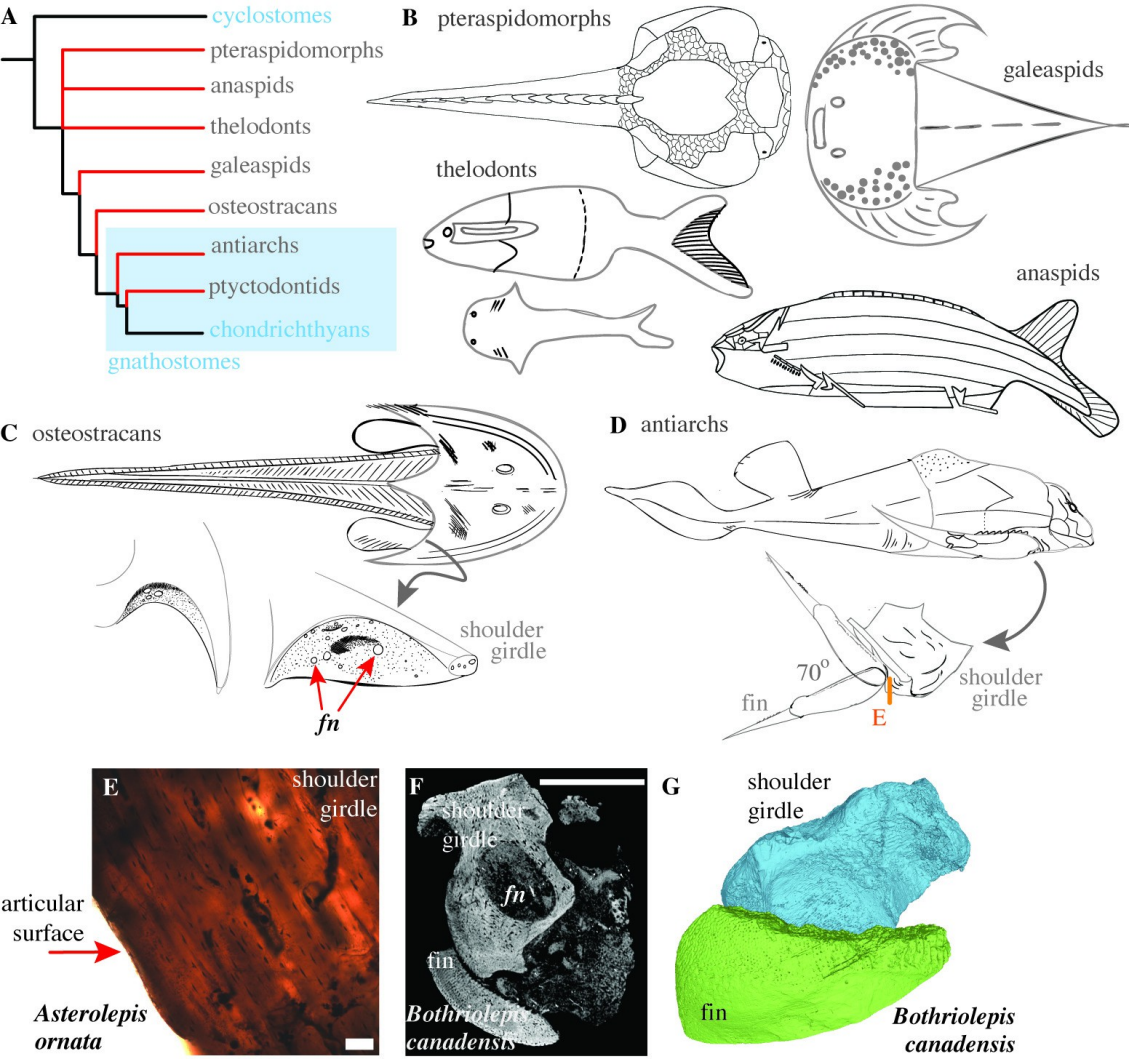
Reciprocally cavitated articulating joints exist in antiarchs. (A) Early vertebrate evolution comprised multiple clades intermediate between cyclostomes and chondrichthyans, now phylogenetically extinct. (B) Simplified morphological representation of a pteraspidomorph heterostracan (*Drepanaspis psammosteid*, adapted from [53]), an anaspid (*Pharyngolepis oblonga*, adapted from [54]), thelodonts (*Loganellia*, top and *Lanarkia*, below adapted from [55]), galeaspid (*Tujiaaspis vividus*, adapted from [56]). (C) External morphology of the osteostracans *Cephalaspis*, the cephalic shield of *Benneviaspis holtedahli* (left) and right zonal part of the cephalic shield in *Cephalaspis ibex* (right) adapted from [57]. The articular surfaces of the pectoral shield show fenestrae (*fn*) for the passage of muscles and nerves. (D) The external morphology of placoderm *Bothriolepis canadensis* and the proposed range of motion at the pectoral fin adapted from [58] (note the location of section for panel E). (E) The histological section of an isolated fossilized shoulder girdle of *Asterolepis ornata*, a closely related and morphologically similar species to *Bothriolepis*, reveals osteocytes and bony tissue. The clean edge in the histological section (red arrow) is the presumptive bony articular surface that suggests that articular cartilage did not lay within the joint. (F) A virtual section from the μCT scan of an antiarch, *Bothriolepis canadensis*, reveals clearly delimited reciprocally articulating surfaces and a joint cavity between the shoulder girdle and the fin. (G) A partial 3D reconstruction of the shoulder girdle and the fin from the μCT scan of the pectoral girdle articulation of *Bothriolepis canadensis* (same specimen as F). The data underlying panels F and G can be found in Morphosource ID: 000663659. *fn*, fenestrae. Scale bars: E, 200 μm; F, 5 mm.

We show that elasmobranch joints exhibit synovial morphology, but our results do not support their presence in cyclostomes, thus placing the evolutionary origin of synovial joints at the common ancestor of extant jawed vertebrates. However, early vertebrate evolution comprises multiple extinct groups that are phylogenetically nested between cyclostomes and chondrichthyans [26] (Fig 8A). Extrapolating whether an extinct group possessed synovial-like morphology is difficult without soft-tissue preservation, requiring inference based on morphology and histology. Therefore, we analyze fossils of the extinct lineages along the gnathostome stem morphologically and paleohistologically.

In the literature, we do not find evidence of cavitated and reciprocally articulating elements in the fossils of early jawless vertebrate fossils belonging to pteraspidomorphs, anaspids, thelodonts, and galeaspids (Fig 8B) [53–56]. They are only known to have head plates formed either of acellular bone or calcified cartilage that were likely interconnected by sutural joints. Compared to other jawless vertebrates, some osteostracans possessed paired pectoral appendages made up of calcified cartilage. It is currently a matter of debate whether their pectoral fins formed an articulation with the dermal head shield [57,59–61]. Well-preserved osteostracan specimens reveal that the region of the head shield that would be proximal to the fin had multiple canals and fenestrae (Fig 8C), suggesting a passage for muscles and nerves to the pectoral fin (see Fig 5, 16, 17, 18 in [57,62]). We propose that the pectoral area with fenestrae could not have functioned as an articular surface with the endoskeletal fin because the joint cavity would be filled with muscles and nerves, preventing the articular surfaces of the pectoral girdle and fin from making close contact or sliding relative to each other. Furthermore, the synovial fluid helps bear joint loads and provides lubrication when compressed inside the joint capsule. The fenestrae in the pectoral shield would cause the synovial fluid to drain under compression, hampering lubrication and the ability to sustain joint loads. Therefore, we conclude that the osteostracan fin likely did not directly articulate with the head shield with a cavitated or a synovial joint.

To constrain the origin of cavitated joints, we analyzed a stem gnathostome, the antiarch placoderm *Bothriolepis canadensis* by μCT scanning a well-preserved and articulated specimen (Fig 8D). Clearly delimited reciprocally articulating surfaces lie between the pectoral girdle made up of cellular bone (osteocytic) and the fin in placoderms (Fig 8F and 8G), as also proposed by previous studies [58,63,64]. The pectoral girdle has fenestrae that likely act as a passage for muscles, vascular tissue, and nerves (Fig 8F). However, they travel inside the hollow fin in antiarch placoderms in contrast with osteostracans where the vascularization is assumed to be external to the cartilaginous fin [62,65]. Thus, the articular surfaces in antiarchs were separate from the fenestrae, and their articular cavity could support compressive loads and function by relative sliding. Whether the articular surfaces were associated with hyaline cartilage or the joint cavity was filled with synovial fluid remains unknown. We note that the histological sections of the bony pectoral girdle show a clean edge at the presumptive articular surfaces (Fig 8E, red arrow), suggesting that articular cartilage did not lay within the joint and that a cavity existed between the pectoral girdle and the fin. However, whether hyaline cartilage formed a separate layer between the two articulating bones, similar to tetrapod jaw joints [25], remains unknown. We hypothesize that antiarchs joints functioned by sliding bony elements with a set of reciprocally cavitated articular surfaces, similar to synovial joints.

The placoderm skeleton is largely formed of dermal bones [66], but recent evidence also points to an endochondral skeleton in the neurocranium beneath the dermal skeleton [67]. For the development of cavitated joints in the dermal pectoral girdle of antiarch placoderms, we propose that separate mesenchymal condensations gave rise to reciprocally cavitated articulations between the pectoral girdle and the fins, and the cells in the middle of the two condensations gave rise to the joint tissue, similar to joint morphogenesis in dermal bone. The joint tissue in their cavitated joints could have been derived from endochondral cartilage [67], but whether the presumptive joint tissue had endochondral origins remains unknown.

## Conclusion

**Fig 9:**
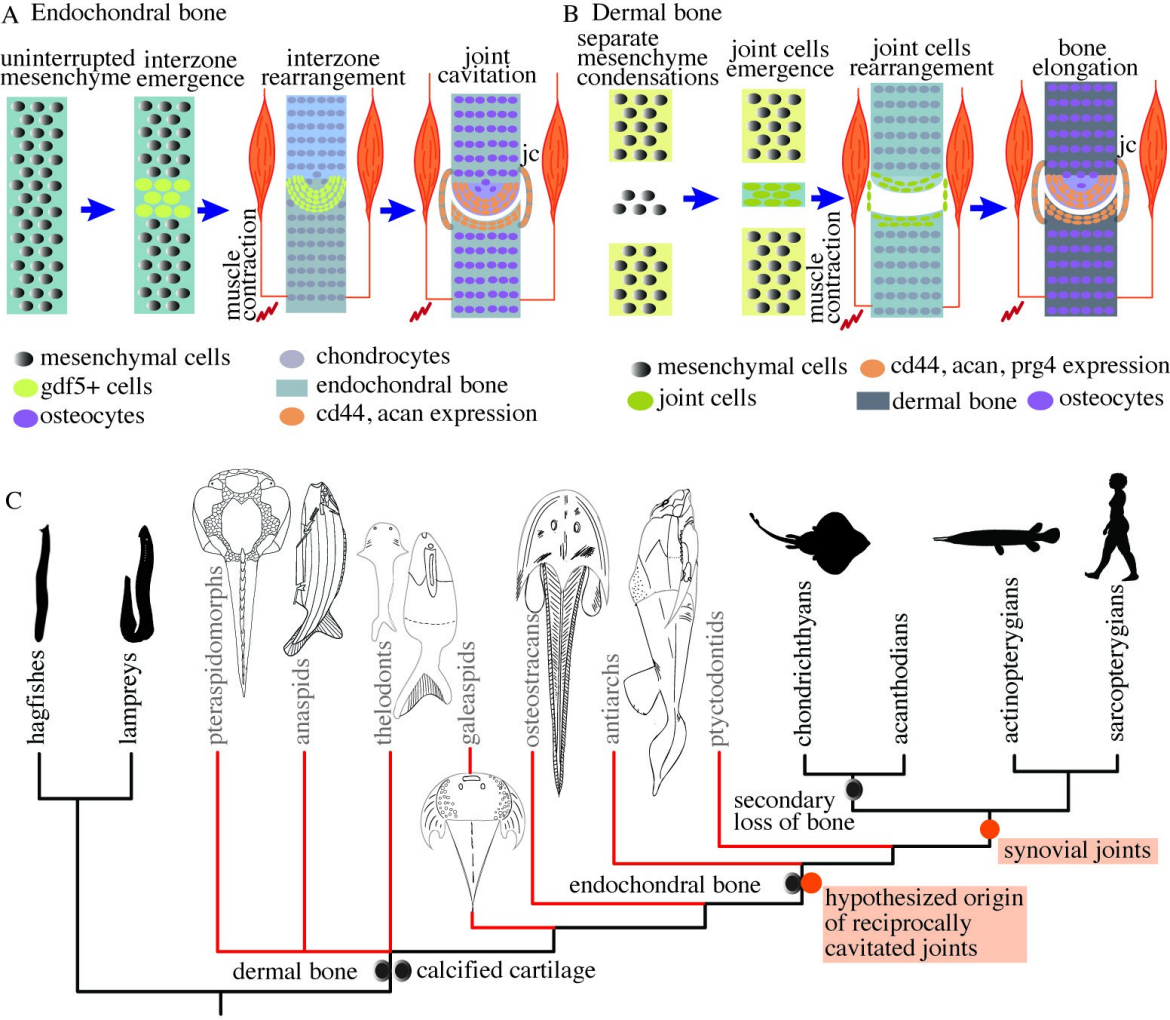
Synovial joints are present in chondrichthyans and reciprocally cavitated joints are hypothesized to have evolved in stem gnathostomes. (A) A simplified pictorial representation of the key mechanisms required for the morphogenesis of synovial joints in the appendicular skeleton of the chondrichthyans. Joint development occurs by the emergence of interzone in the uninterrupted mesenchyme that cavitates to form a joint cavity. TGF-*β* and Wnt signaling molecules are expressed, and muscle contraction is required for joint cavitation. (B) A simplified pictorial representation of the mechanisms involved in the morphogenesis of synovial joints in the dermal skeleton. Joint development occurs through the emergence of an independent mesenchymal condensations that develop into dermal bones. The bones could form either through intramembranous ossification (no intermediary cartilage) or perichondral ossification (via secondary cartilage). Joint cells arise between the two developing bones and form the joint tissue. (C) Synovial joints exist in extant jawed vertebrates (gnathostomes), but our results do not support their existence in cyclostomes. The presence of reciprocally shaped and cavitated joints in the dermal skeleton of antiarchs suggests that joints that function by relative sliding (similar to synovial joints) first originated in stem gnathostomes. Phylogenetic tree adapted from Donoghue and Keating [26]. jc, joint capsule.

Our results show that chondrichthyan joints are morphologically and developmentally similar to the synovial joints in the endochondral skeleton of the osteichthyans, including tetrapods (Fig 9A). We were unable to identify any reciprocally cavitated joints in lamprey and hagfish. Thus, we conclude that synovial joints are a shared feature of extant jawed vertebrates (Fig 9C). Furthermore, we identify the earliest reciprocal and cavitated joint morphology in vertebrates in an antiarch placoderm and propose that their cavitated joints followed the developmental processes similar to those in extant dermal skeletons (Fig 9B). We infer that cavitated joints with reciprocally articulating surfaces that functioned by sliding first appeared in the bony dermal skeleton of gnathostomes (Fig 9C).

The expression of proteins from TGF-*β* and Wnt signaling pathways, such as *Gdf5* and *β*-catenin, in the little skate embryos during joint morphogenesis is similar to their expression during synovial joint development in tetrapods. Further pharmacological studies are required to reveal the roles played by Wnt and TGF-*β* signaling in the development of joints in chondrichthyans. In sea lamprey, *Gdf* is expressed in the pharyngeal endoderm and mucocartilage of the ventral pharynx, and *Bapx* in the epidermis of the ventral pharynx [68]. If further studies reveal that lampreys also express and require *Gdf5* at the site of presumptive cartilaginous joints, it would suggest a conserved role of TGF-*β* pathway in the development of cartilaginous and synovial joints in the endochondral skeleton of vertebrates. The expression of proteoglycans and GAGs in the lamprey cartilage [46,47] also suggests their primitive role in regulating osmotic and turgor pressure in the cartilaginous skeleton for countering the mechanical trauma in the absence of mineralization [35], similar to chondrichthyans [43,69]. We hypothesize a co-option model wherein the molecular pathways required for the development and function of synovial joints are derived from the novel recruitment of preexisting TGF-*β* and Wnt signaling pathways and the expression of specific proteoglycans at the articular surfaces. The glycosaminoglycans and collagens in the mineralized as well as non-mineralized cartilaginous skeleton of elasmobranchs provide it with strength and stiffness comparable to trabecular bone [43,69,70]. Thus, the bendability of the elasmobranch skeleton is not superior to bony skeletons. The mechanical properties of lamprey fibrocartilage are comparable to mammalian articular cartilage [71], and therefore, it is comparatively softer than chondrichthyan cartilage. Lamprey cartilage also expresses lamprin, an elastin-like protein, in their nasal, branchial, and pericardial cartilage, imparting enhanced skeletal flexibility [72]. Our results are consistent with such functional studies showing that chondrichthyans, like tetrapods, rely on synovial joints for exhibiting considerable jaw and fin movements in their stiff skeleton that reaches several meters in size, but with the exception of the rest of the vertebrates, our results do not support their presence in cyclostomes.

Studies suggest that the endochondral bone first appeared in the neurocranium of placoderms [67]. However, no reciprocally articulating elements have been identified in the endochondral skeleton of the fossil taxa nested between placoderms and chondrichthyans. Synovial joint morphogenesis in the chondrichthyan skeleton relies on developmental processes similar to those observed in the endochondral appendicular skeleton in tetrapods, minimally placing the evolution of synovial joints in the endochondral skeleton at the common ancestor of extant gnathostomes. However, we cannot conclusively rule out the presence of endochondral synovial joints in other stem gnathostomes due to a paucity of well-preserved and articulated specimens. Our study raises questions about the early evolution of synovial joints in dermal and endochondral skeletons, and the evolution of separate developmental processes required for their morphogenesis.

## Materials and methods

### Animal procurement, care, and procedures

The Institutional Animal Care and Use Committee (IACUC) of the University of Chicago approved the procedures on the little skate and sea lamprey (ACUP# 71033). All procedures were performed at the University of Chicago. The little skates and bamboo sharks were obtained from the Marine Resources Center, Marine Biological Laboratory, Woods Hole, Massachusetts, United States of America. For little skates, we followed the developmental staging scheme from Maxwell and colleagues [27] and used animals from stages 32 (27 to 29 weeks old) and 33 (*>*29 weeks old). Juvenile sea lampreys were obtained from Acme Lamprey (Harrison, Maine, USA), ß and adult sea lampreys were procured from John G. Shedd Aquarium (Chicago, Illinois, USA). For μCT scanning, histology, skeletal staining, immunostaining, in situ hybridization, and after the completion of the muscle paralysis experiments, the animals were euthanized using 0.5% tricaine methanesulfonate (MS-222, Syndel) until the cessation of heartbeat and fixed in 4% PFA (Acros Organics, Item No. EW-88353-82) overnight (embryonic skates), or for 4 days (juvenile sea lamprey and skates) before transferring them to 1X PBS (Fisher Bioreagents).

### Contrast-enhanced μCT scanning

Scanning was performed on Phoenix v*|*tome*|*x S 240 from GE (PaleoCT facility, RRID: SCR024763, University of Chicago) using the 180 kV nano-focus tube. Sea lamprey was stained using Ruthenium red (Cayman Chemical, CAS Number 11103-72-3) to give rise to contrast between cartilage and muscles following the protocol of Gabner and colleagues [73] with the modification that the juvenile lampreys were kept in the staining solution for 7 days. The scanning parameters were 80 kV of Voltage, 180 μA beam current, and images were generated with a voxel size of 14.238 μm. The little skate embryo (stage 33), adult little skate, and hagfish (*Myxine glutinosa*) head were stained in phosphomolybdic acid (PMA, Sigma Aldrich). Specimens were stepped in 25%, 50%, and 75% solutions of sucrose (Sigma Aldrich) in 1× PBS for 1 h each with shaking, and then transferred to 20% sucrose in PBS overnight. The specimens were transferred to 5% PMA solution in 1× PBS for a week before scanning. Two scans were performed for the little skate, one focused on the pelvis and another one of the whole embryo. The scanning parameters used were 90 kV Voltage, 270 μA beam current, and the images were generated with a voxel size of 8.738 μm for the pelvis, and 100 kV Voltage, 135 μA beam current, and a voxel size of 26.181 μm for the whole embryo. For the adult skate, the scanning parameters used were 100 kV Voltage, 300 μA beam current, and the images were generated with a voxel size of 38.856 μm for the jaw and 37.831 μm for the pelvis. For adult hagfish, the scanning parameters used were 60 kV Voltage, 380 μA beam current, and the images were generated with a voxel size of 31.763 μm. For adult lamprey stained for 5 days in 1% iodine-potassium iodide, the scanning parameters used were 100 kV Voltage, 80 μA beam current, and the images were generated with a voxel size of 17.029 μm. The fossil of placoderm, *Bothriolepis canadensis* (MB.F.9200.a, Museum für Naturekunde Berlin), was scanned using 80 kV Voltage, 200 *μ*A beam current, and the images were generated with a voxel size of 13.144 μm. Segmentation and reconstruction were performed on Amira 3D 2021.1 (FEI SAS, Thermo Fisher Scientific, Hillboro, Oregon, USA). MeshLab v2023.12 [74] was used for visualization.

### Histochemical staining

The embryonic and juvenile little skates (*Leucoraja erinacea*), juvenile and adult sea lamprey (*Petromyozon marinus*), bamboo shark (*Chiloscyllium plagiosum*), and chicken (*Gallus gallus*) were stepped in 70% ethanol and embedded in paraffin. For staging the chicken embryos, we followed the developmental series proposed by Hamburger and Hamilton [75]. Paraffin embedding was performed by the Human Tissue Resource Center at the University of Chicago. The paraffin-embedded blocks were sectioned at 10 μm using a rotary microtome (HM 330, Microm Heidelberg). Sections were mounted on TOMO IHC adhesive glass slides (TOM-1190, Matsunami). HE staining was performed using Hematoxylin (Sigma-Aldrich) and Eosin (Sigma-Aldrich) following the protocol of Gillis and colleagues [76]. Safranin-O staining was performed using Safranine-O (Fisher Scientific), Hematoxylin, and Fast Green FCF (Sigma Life Science). The sections were dewaxed and hydrated to distilled water, stained with Weigert’s iron hematoxylin working solution for 10 min, washed in running tap water for 10 min, stained with fast green (FCF) solution for 5 min, rinsed in 1% acetic acid solution for 15 s, stained with 0.1% safranin O solution for 5 min, and stepwise dehydrated in increasing concentrations of ethanol and finally, histosol. Picrosirius staining was performed using Direct Red 80 (Sigma Aldrich). Slides were dewaxed and hydrated to distilled water, stained with Weigert’s iron hematoxylin working solution for 10 min, washed in running tap water for 10 min, stained in picrosirius red solution (0.1% Direct Red 80 in saturated aqueous solution of picric acid) for an hour, washed in 0.5% acetic acid solution, and dehydrated in 100% ethanol and histosol. Slide sections were mounted using Permount (FisherChemicals, Fisher Scientific).

Whole-mount alcian blue and alizarin red skeletal staining was performed on the embryos of little skates (*Leucoraja erinacea*). After euthanasia and fixation, samples were stepped in 70% ethanol, followed by submersion for 2 h in acetic ethanol (30 ml glacial acetic acid, 70 ml absolute ethanol), and then stained overnight using alcian blue solution (0.02% alcian blue in acetic ethanol). Samples were destained overnight in acetic ethanol and stepped into distilled water, followed by overnight staining with Alizarin red solution (0.1% alizarin red in 1% KOH solution). The animals were soaked in 1% trypsin in 2% sodium tetraborate until clear, and transferred into 3:1 0.5% KOH:glycerol, and stepped to 80% glycerol. The chemicals used were acetic acid (A6283, Sigma-Aldrich), potassium hydroxide (P250-1, Fisher Scientific), glycerol (Fisher Scientific), alcian blue 8GX (Sigma-Aldrich), Alizarin Sodium Sulfonate (CI 58005, Sigma), sodium tetraborate decahydrate (Sigma-Aldrich), and trypsin (J60402.18, Alfa Aesar).

### Immunofluorescence

Fixed tissues were stepped in 1× PBS overnight and then in 20% sucrose solution prepared in 1× PBS before embedding them in Tissue-Plus OCT compound (Fisher Healthcare). Cryosections were obtained at a thickness of 10 μm using Leica CM3050 S. Apart from collagen-II immunostaining that was performed on paraffin sections, immunostaining for aggrecan, CD44, aggrecan, and *β*-catenin was performed on cryosections. Paraffin sections of thickness 10 μm were prepared as described above. Collagen-II immunostaining on paraffin sections was performed using the protocol of Gillis and colleagues [77]. Aggrecan, CD44, and *β*-catenin immunostaining was performed by washing the cryosections in 1× PBS for 5 min, blocking them in blocking buffer (5% goat serum in PBT prepared with 0.1% Tween-20, Sigma Aldrich) for an hour at room temperature followed by overnight primary antibody incubation at 4°C. Primary antibody was washed 3 times in 1× PBS for 5 min each, followed by overnight incubation in secondary antibody, washed 3 times in 1× PBS for 5 min each the next day followed by mounting. Primary and secondary antibodies were diluted in blocking buffer. The following primary antibodies were used: Collagen-II (II.II6B3, Developmental Studies Hybridoma Bank, University of Iowa; 1:20), *β*-Catenin (ProteinTech, 17565-1-AP, 1:300), Aggrecan (12/21/1-C-6, Developmental Studies Hybridoma Bank, University of Iowa; 1:20), Hyaluronic acid receptor (H4C4, Gene name: CD44, Developmental Studies Hybridoma Bank, University of Iowa; 1:20). The following secondary antibodies were used at a dilution of 1:300: Cy3 donkey-anti-rabbit (Jackson, 711-166-152), Cy5 donkey-anti-mouse (Jackson, 715-175-151), CF 633 goat-anti-mouse (Biotium, 20120–1), CF633 goat-anti-rabbit (Biotium, 20122–1), and Cy3 donkey-anti-mouse (Jackson). The slides were mounted using Fluoromont-G with DAPI (Invitrogen). All immunostaining was carried out in quadruplets and the controls were prepared by following the same protocol but in the absence of the primary antibody. Individual panels and gradient plots are shown in S1–S4 Figs.

### mRNA in situ hybridization

Probes were designed for *Petromyzon marinus* for hyaluronic acid and proteoglycan link protein 3 (HAPLN3), using the sequences from the National Library of Medicine, National Center for Biotechnology Information (GenBank XM032975677). For designing the probe, we amplified the cDNA of the sea lamprey using CCGAATAGTGTGGTCAGGGT as the forward primer and TGCGGCTTCATTTCATACGG as the reverse primer. The fixed juvenile sea lamprey was stepped in 100% methanol and kept at −20°C before it was paraffin-embedded and sliced by a microtome into a thickness of 10 μm sections. In situ hybridization was performed on the paraffin sections following the protocol by Sugahara and colleagues [78] with the modifications that we used 10 μg/ml of proteinase-K (Thermo Scientific) for 10 min for the digestion of sections, Nuclear Fast Red (Newcomer Supply) for counterstaining after the color reaction, and Permount (FisherChemicals, Fisher Scientific) as a mounting medium.

For in situ hybridization in the embryos of little skates for analyzing the expression of growth differentiation factor 5 (gdf5), we selected the sequence available for *Amblyraja radiata* (GenBank XP032897637) and performed a nucleotide BLAST against the little skate transcriptomic contigs [79,80] and found a high degree of similarity with contig 44189 (bit score = 383, identity = 215/217 with a query length of 817, similarity = 99, e-value = 2e-105). We designed probes for contig 44189 by amplifying the cDNA of the little skate using TGCACTCTCCGATTGTCCAA as the forward primer and GCAACCACAGGACTCTACCA as the reverse primer. The fixed skate embryos were stepped in 100% methanol and stored at −20°C before paraffin embedding. For in situ hybridization on the paraffin sections, we followed the protocol in [81] with modifications according to [33,77].

### Muscle paralysis

Little skates from the embryonic stages 32 were raised in tanks after removing their egg cases. Two groups were maintained: paralyzed (*n* = 3) and control (*n* = 3). We maintained water oxygenation in the tanks using air pumps, ensuring consistent aeration and water quality. Water temperature was maintained at 17°C. The experimental tank consisted of 150 ml of water mixed with 0.01 grams of tricaine methanesulfonate (MS-222) and 4.5 grams of sea salt (Instant Ocean, Spectrum Brands Pet LLC, Blacksburg, VA) (0.007% tricaine, 3% instant ocean). The control tank contained 150 ml of water and 4.5 grams of sea salt but no tricaine methanesulfonate. We replaced the water in both tanks every 2 days. The embryos were raised for 18 days for both the control and the experiment conditions as it takes around 3 weeks for the little skates from embryonic stage 32 to cavitate their joints. After the completion of the experiment, we euthanized and fixed the embryos, as described above. We performed histology on the paraffin sections with Hematoxylin, Eosin, and Safranin-O, and immunostaining for *β*-catenin.

### Fossil procurement and microstructural analysis

The fossil of *Asterolepis ornata* (MB.F.1786.15) was obtained from Museum für Naturekunde, Berlin, Germany, and approval was obtained for destructive sampling. For the microstructural analysis of fossils *Asterolepis ornata*, the slides containing sections were prepared at the Paleohistology lab at Field Museum (Chicago, Illinois). Fossils were embedded into epoxy overnight (Amazing Clear Cast Epoxy, Alumilite Corporation, Polytex Dev. Corp. Brand). The embedded fossils were cut to expose the cross-section of interest, polished, and glued on glass slides. Ecomet 5 (Buehler Ltd., Lake Bluff, Illinois, USA) was used for the subsequent grinding and polishing of the fossil surfaces.

### Microscopy and image analysis

Microscopy for histochemical staining was performed on Axioskop 2 mounted with Zeiss Calibri 7 camera and a polarizer. The software used for image acquisition was Zen 3.8 (Carl Zeiss Microscopy GmbH). Whole mount specimens were imaged on Leica DFC425 C and the software used for image acquisition was AmScope x64 (4.11.20896.20220521). Immunostaining slides were imaged at Zeiss LSM 710 Axio Imager 2 (Release Version 8.1.0.484, Carl Zeiss Microscopy GmbH) and the software used was Zen 2012. ImageJ2 Version 2.14.0/1.54f and Adobe Illustrator 28.0 (Adobe, San Jose, California, USA) were used for processing images and constructing the plates, respectively.

## Supporting information

S1 Fig Expression of collagen-II in embryonic stage 32 and stage 33 little skates visualized using a grayscale plot for individual channels. (TIF)

S2 Fig Expression of aggrecan in juvenile skate, and in embryonic stage 32 and stage 33 little skates visualized using a grayscale plot for individual channels and a gradient plot to resolve and compare the intensity of expression. (TIF)

S3 Fig Expression of CD44 in juvenile skate and in embryonic stage 32 and stage 33 little skates visualized using a grayscale plot for individual channels and a gradient plot to resolve and compare the intensity of expression. (TIF)

S4 Fig Expression of *β*-Catenin in stage 32 and stage 33 little skate embryos visualized using a grayscale plot for the individual channels. (TIF)
