# Supplementary figures and images for: Synovial joints were present in the common ancestor of jawed fish but lacking in jawless fish
Source: PLoS Biol. 2025 Feb 25;23(2):e3002990. doi: 10.1371/journal.pbio.3002990 (PMC11856278; doi:10.1371/journal.pbio.3002990)

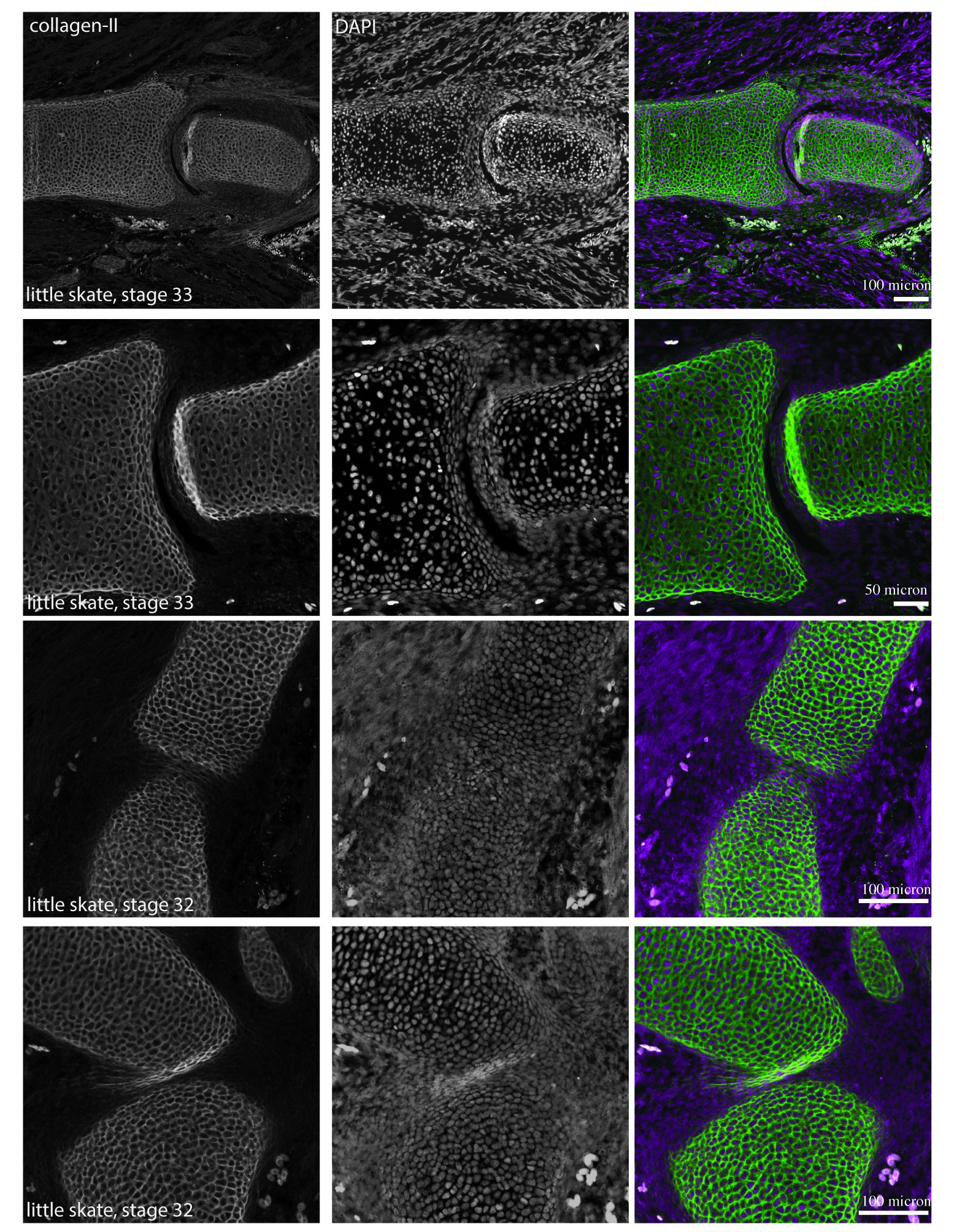

Supplement: S1 Fig — (TIF) [file pbio.3002990.s001.tif]

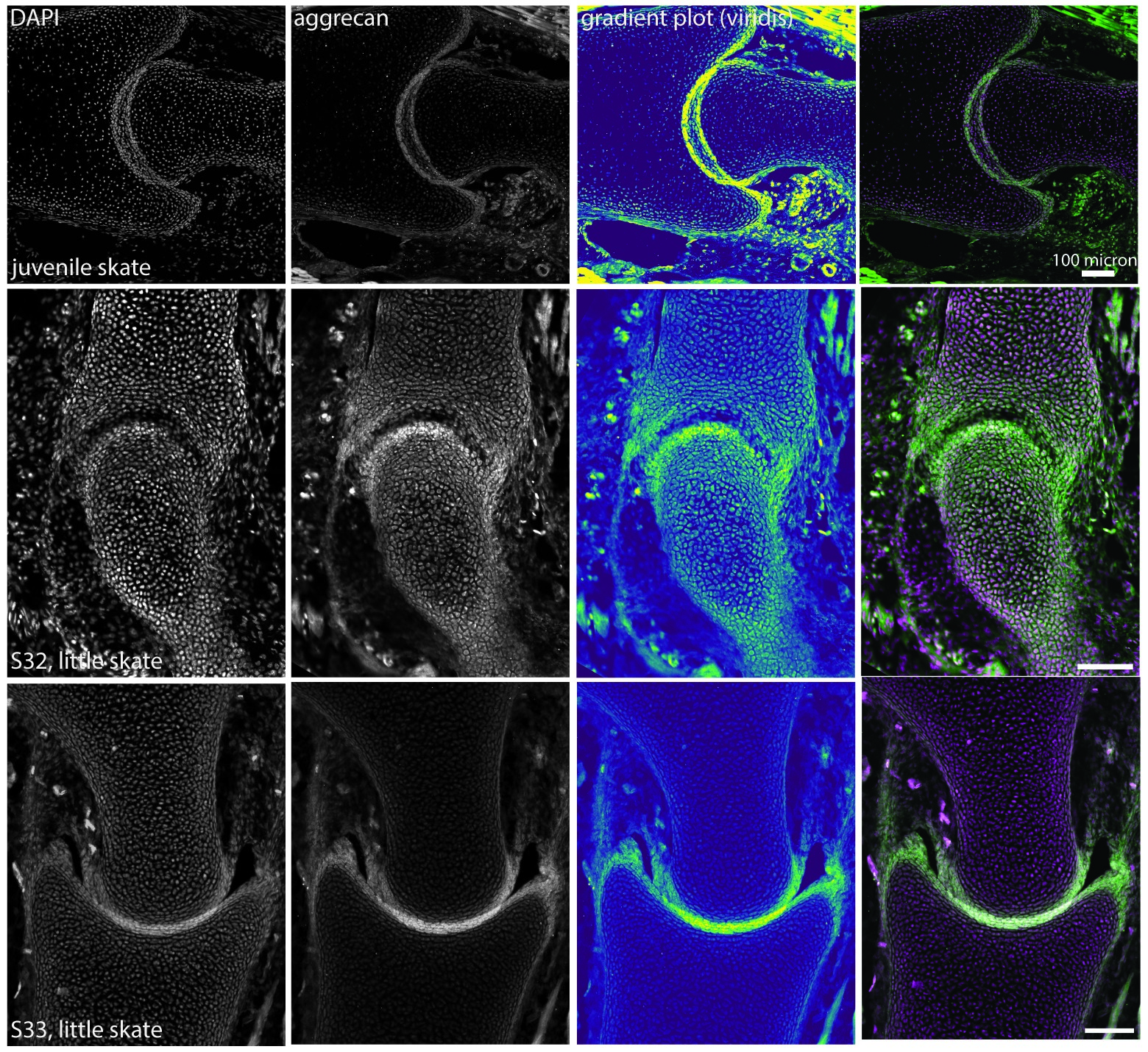

Supplement: S2 Fig — (TIF) [file pbio.3002990.s002.tif]

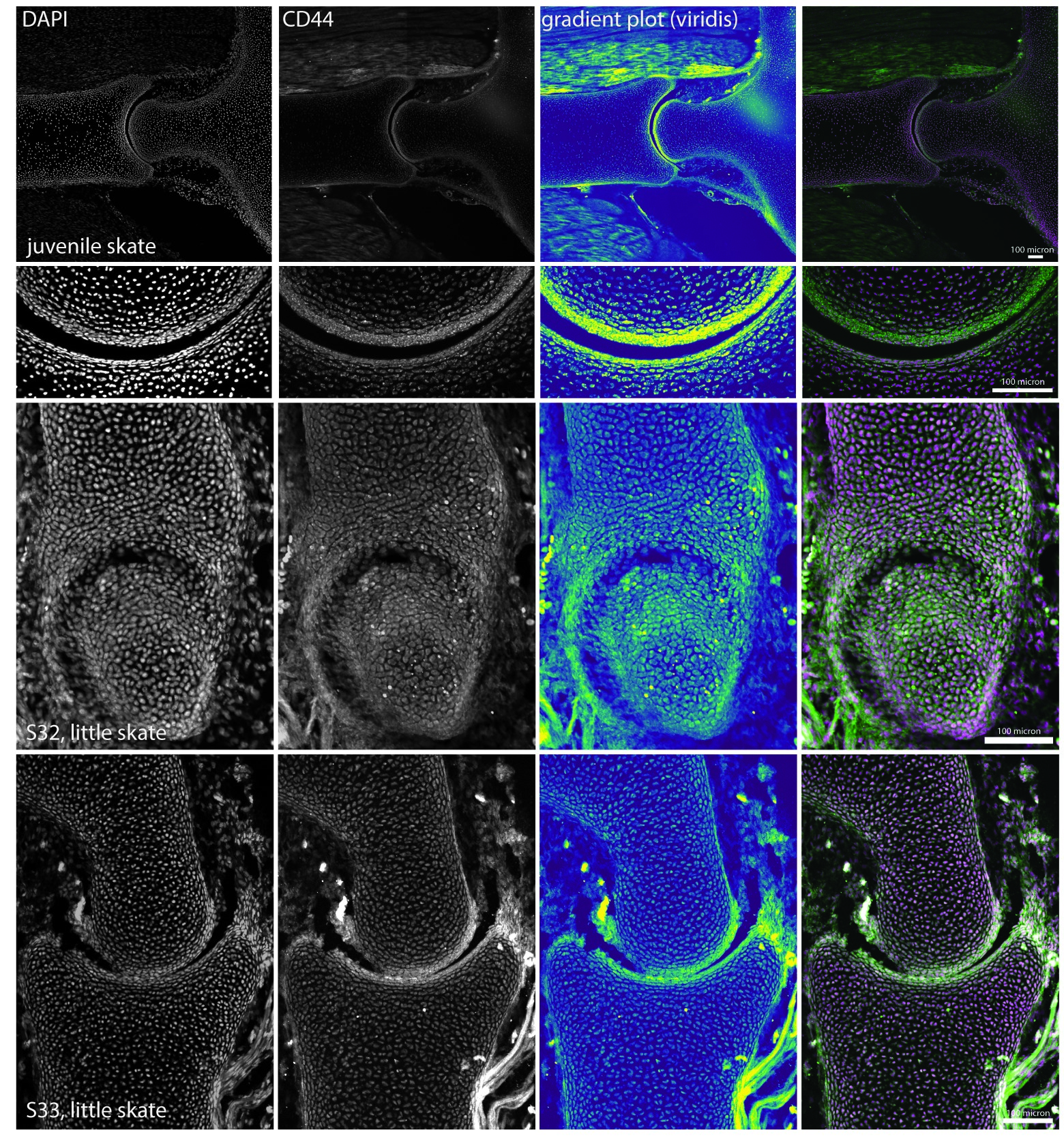

Supplement: S3 Fig — (TIF) [file pbio.3002990.s003.tif]

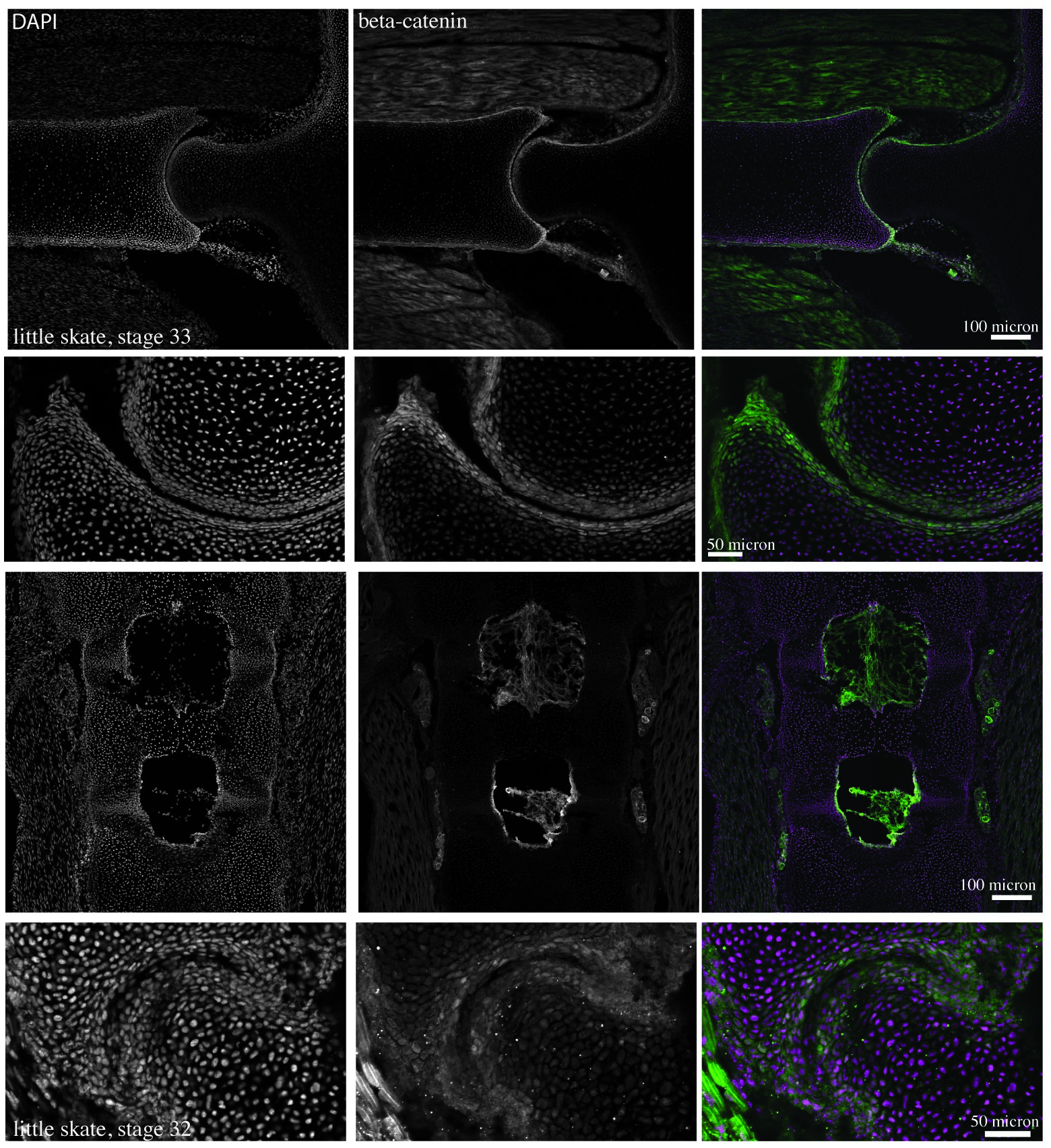

Supplement: S4 Fig — (TIF) [file pbio.3002990.s004.tif]
